# The Challenge to Our Community: Openness, Relevance, Trust

**DOI:** 10.3389/frma.2019.00005

**Published:** 2020-02-26

**Authors:** Caroline S. Wagner

**Affiliations:** Glenn College, The Ohio State University, Columbus, OH, United States

**Keywords:** openness, open access, policymakers, scholarly academics, indexing, policy, trust

*Research Policy and Strategic Management*, a new section of *Frontiers in Research Metrics and Analytics*, begins publication with great optimism for the use of the open access format to enhance readership, rigor, and dialogue for the community of scholars and policymakers.

*Research Policy and Strategic Management* takes advantage of the *Frontiers* platform to accomplish three goals: (1) to facilitate faster publication of policy-oriented publications in an open-access (OA) format; (2) to contribute open evidence, data, and expertise supporting publications in order to aid reproducibility and support policymaking; and (3) to boost trust between policymakers and academia by widening the channels of communication between these communities. Each of these points is expanded below. We have plans to address two clear challenges in OA—the payment of Article Processing Charges (APCs) and the journal indexing—as is discussed below.

## Goal: A Faster, More Open Publication Pipeline

Any scholarly venue must publish excellent scholarship; this will therefore be a primary purpose of *RPSM*, with a focus being on ensuring that evidence-based policy—a bedrock principle of public administration—is served. The print-journal model, effective for over 300 years, is now overtaken by digital modes and venues, moving scholarship into the hands of users at rapid pace. As we know, the print-journal model often requires months to years of review, editing, and publication processing. *RPSM* will speed up this process, and the editors will work to draw the attention of policymakers to the work. Rigor in intellectual contribution will remain a primary goal.

Greater speed and openness are provided through an innovative open-access platform, fully compliant with Plan S^1^. The platform offers better dissemination of academic scholarship and greater capacity to interface with readers. Facilitated by the *Frontiers in* platform, this section of *Frontiers in Research Metrics and Analytics* will publish high-quality research in a timely way, moving scholarship briskly into a public forum and creating a venue for evidence-based policy discussion—all at a pace far accelerated over the current communications processes. We will create and act as a supportive community.

## Goal: Greater Interactions with Readers

Second, the new venue will provide a discussion function to accompany scholarly work. This responds more to a threat than an opportunity, also tied to new media. In an era when, paradoxically, freer, ubiquitous communication has empowered those who wish to spread their ideas to decision makers, some are manipulating and distorting facts using new media. One way to address this problem is to create tighter links between those producing scholarship and those who need research. Some forces in society seek to use media to obscure or blur information in order to undermine authority and introduce distrust—to move beyond “truth” or “falsehood” into a deep uncertainty where nobody knows what is real. At that point, no expert or policymaker can be trusted or believed. In such an uncertain space, it is impossible to evaluate and debate facts. Thus, in scholarship, we see a call to “tear down the journal system”—as if it is a bastion of some “ancient regime” that embodies an entrenched and illicit meritocracy rather than a system that offers an expert filter to judge the believability of claims. This dynamic cries out for forums that allow questioning but provide verification. *Frontiers in Research Metrics and Analytics* is an open platform that will aim to close that gap.

## Goal: Build Trust in Expertise

The publication will be open access, with open-source links to data, authentication of authorship and contributorship, validation of sourcing, and open peer review. Authentication in scholarly communication is increasingly needed in an era of “deepfakes”—predatory publishers, increasing plagiarism, hoaxes, and spoofing, use of unverified data, and an intensifying competition for recognition. Moreover, the publication will be committed to pushing back on the many factors that underprop gender and racial inequality by consciously putting inclusion as a primary goal.

The process of verification of academic research within the policy framework is slow and varied. Academics often choose Research Topics in isolation, not always knowing actual social or policy needs. Their role of “speaking truth to power” is muffled and garbled by the processes of publication and the additional pressures of indicators of “impact” and “quality.” The process of speaking truth—presenting researched evidence—has the goal of confirming or falsifying the statements of or actions of the government, but the “expert” is often called upon simply to prove their own credibility rather than to defend their observation. The process, however, could also be opened up to engage with new kinds of material—material that is open and informed by research—and employ different communication processes that present and socialize the production of evidence, integrate science with valued sensibilities, and work across and bring together different viewpoints. This is one of the goals of the new outlet, and it is enabled by this new, highly functional platform.

## Challenge: Article Processing Charges (APCs)

Open access itself is not the golden door to an equitable landscape for academic researchers—it only offers a solution to the problem of limited accessibility of published scholarship. In turn, OA introduces several troublesome features—in particular, article rocessing charges (APCs) and the lack of a sanctioned “impact factor”—whose absences actually stack the deck against younger researchers who do not have funds to pay for publication and who need source-based impact factor indicators and citations to gain promotion. For those researchers who do not have funds to pay for publication, Frontiers has a waiver mechanism to ensure participation regardless of ability to pay.

## Challenge: Indexing Status

*Frontiers in Research Metrics and Analytics* will be indexed in Google Scholar, DOAJ, CrossRef, and CLOCKSS. It will soon be listed in PMCID and Dimensions (based upon DOI). For scholars who may need this help, we will produce a statement qualifying the contribution and impact of their work. This follows a number of recent calls to move beyond metrics and toward what some call “profiles, not metrics”—returning the responsibility to those offering to “measure” scholarly quality (see the Leiden Manifesto)^2^.

Finally, the editorial board is committed to addressing inequities in the current system that impact gender and geographic representation. We are committed to openness in all ways. The openness of the format allows us to address these problems from the ground up as we organize. The board of editors and reviewers will be gender balanced and broadly inclusive. We will keep this as a goal in choosing other peer reviewers. In contrast to existing publication schemes, we seek to create a supportive community that enhances its members and creates new knowledge that serves society.

## Author Contributions

The author confirms being the sole contributor of this work and has approved it for publication.

### Conflict of Interest

The author declares that the research was conducted in the absence of any commercial or financial relationships that could be construed as a potential conflict of interest.

## Footnotes

^1^https://www.coalition-s.org/

^2^http://www.leidenmanifesto.org/

